# Effects of Waterpipe Tobacco Smoking on the Spirometric Profile of University Students in Palestine: A Cross-Sectional Study

**DOI:** 10.1155/2020/5949834

**Published:** 2020-06-27

**Authors:** Zaher Nazzal, Deema Odeh, Fatima Azahraa Haddad, Mohammad Berawi, Belal Rahhal, Hasan Yamin

**Affiliations:** ^1^Division of Family and Community Medicine, Faculty of Medicine and Health Sciences, An-Najah National University, Nablus, State of Palestine; ^2^Department of Medicine, Faculty of Medicine and Health Sciences, An-Najah National University, Nablus, State of Palestine; ^3^Division of Physiology, Pharmacology and Toxicology, An-Najah National University, Nablus, State of Palestine; ^4^Al Hakim Medical Complex, Nablus, State of Palestine

## Abstract

**Background:**

Waterpipe smoking in young individuals is increasing with limited studies addressing its respiratory health effects. The aim of the study was to determine the effect of waterpipe smoking on young adults' lung functions. Spirometric parameters were compared between waterpipe smokers and nonsmokers.

**Methods:**

A comparative cross-sectional study of university students, including males and females, was conducted. An interviewer-administered questionnaire was used to record students' characteristics. The spirometry test was performed to assess students' lung functions; we recorded the forced expiratory volume in the first second (FEV1), forced vital capacity (FVC), FEV1/FVC ratio, peak expiratory flow (PEF), and forced expiratory flow between 25 and 75% of FVC (FEF_25–75_%).

**Results:**

A total of 300 apparently healthy students (150 waterpipe smokers and 150 nonsmokers) were included in the study. Waterpipe smokers showed significantly lower values in FEV_1_, FEV_1_/FVC ratio, PEF, and FEF_25–75_% compared to the nonsmoker group (*P* < 0.05 to *P* < 0.001). The subgroup analysis on female students (50 WP smokers and 50 nonsmokers) showed a significant decrease in FEV_1_/FVC ratio, PEF, and FEF_25–75_% parameters (*P* < 0.001).

**Conclusion:**

Waterpipe smoking is associated with reduced spirometric parameters in healthy young adults with relatively limited smoking years.

## 1. Introduction

Waterpipe smoking (WPS) is an old tobacco smoking method dating back centuries. In the last two decades, however, there has been a global increase in the prevalence of WPS, particularly among the youth [1, 2]. WPS is perceived by its new generation of users as a less harmful alternative to cigarette smoking [3, 4]. This widespread misconception is attributed to the way smoke moves through water in the waterpipe (WP) device, giving a false sense of being filtered. Other factors favoring this misconception are the use of flavored and aromatic tobacco and the essential part it plays in social gatherings and cafe culture [5]. With this worryingly increasing popularity, WPS has been recently described as an emerging health crisis [6].

Pulmonary function tests (PFTs) are valuable measures to assess function. Spirometry, as a part of PFTs, is an important tool in the investigation and monitoring of general respiratory health. It provides measurements of lung volumes and flow rates. Parameters are recorded as forced expiratory volume in the first second (FEV_1_), forced vital capacity (FVC), FEV_1_/FVC ratio, peak expiratory flow (PEF), and forced expiratory flow between 25 and 75% of FVC (FEF_25–75_%) [7].

WPS hazardous effects on the respiratory system have been previously studied,pParticularly those related to spirometry, which showed a significant decrease in many parameters [8]. Most of the previous work regarding WPS focused on the middle- and old-aged where long exposure to WPS has taken its effect [8, 9]. Nevertheless, in-depth understanding of the effects of WPS on the youth is crucial and remains limited to a few studies [10, 11].

A study by Moe et al. on Saudi male young adults (mean age 21.4 years) reported a significant decrease in several lung function parameters among WP smokers relative to their control group [11]. Hawari et al. compared the pulmonary function of 69 male WPS to 69 nonsmokers (mean age = 22.1 years). WP smokers demonstrated significantly lower values of FEV1, FVC, PEF, and TLC [10]. Another study that took place in Iran, including male and female adults (mean age = 40 ± 12.2 years), showed that all spirometric values in 58 WP smokers were lower as compared to 50 nonsmokers [12].

As of 2014, WPS prevalence was shown to be significant among university students in Palestine. About one-fourth of the students were identified as regular WP smokers, noticeably starting smoking at a young age [13, 14]. Interestingly, Palestinian female students are much more likely to smoke waterpipe than cigarettes [14]. It appears to be more socially acceptable than cigarette smoking and is perceived as less harmful [4].

Herein, we are exclusively testing young adults, both males and females, who are presumed to have better lung compliance and less smoking years. To our knowledge, this is the first study to include young female participants. This case is especially important in Palestine where WPS has a long history of popularity and is available everywhere with no age restrictions. The results of this study will hopefully extend available knowledge on WPS in Palestine and lay the ground for health promotion programs and tighter policy control. Most tobacco policies are cigarette-tailored, and adapting them to waterpipe specifications is crucial in terms of health warnings, youth access limitation, bans on advertisement and flavoring, and clean indoor air laws. Furthermore, physicians and health educators in Palestine and in the region can use these results further to educate patients on WPS harms and encouraging cessation. The aim of this study is to assess the pulmonary function among WPS university students and to compare their spirometric profiles to nonsmoker students.

## 2. Methodology

### 2.1. Study Design and Population

This study is a comparative cross-sectional study spread over 4 months, from November 2019 to February 2020. It was approved by the institutional review board (IRB) at the An-Najah National University. Written informed consent was obtained from all participants.

We calculated the sample size by the package program EPI INFO. It was based on a 95% level of confidence interval and 80% power of the study. We considered the expected mean of FEF 25–75% to be 99 ± 20 in the WP smoker group and 106 ± 24 for the nonsmoker group. These values were considered based on the results of similar studies [9, 10]. We ended up with a total sample size of 300: 150 in the WP smoker group and 150 in the nonsmoker group.

A convenience sampling technique was used to invite the study participants. As such, participants were approached and recruited from the university campuses by asking them to join voluntarily, after being introduced about the study. Responses to recruitment were generally positive with approximately 20% who opted not to join, mostly for having no time. All students who were apparently healthy and either never used tobacco (never-smokers) or regular waterpipe smokers who smoked at least once a week for at least one year, with no other forms of tobacco used [15], were invited to join the study. The following exclusion criteria were applied: cigarette smoking, having a chronic respiratory illness; recent respiratory tract infection; the presence of comorbidities (cardiovascular diseases, neoplasia); body mass index (BMI) of 40.0 kg/m^2^ or more; current pregnancy; or imperfect performance of the requested respiratory maneuvers.

### 2.2. Procedures and Data Collection

A CareFusion MicroLab ML3500 MK8 Spirometer was used to evaluate the lung function. It was compliant with the American Thoracic Society (ATS) and European Respiratory Society (ERS) 2005 Standardization of Spirometry, with an accuracy of ±3%. The spirometry testing was performed in accordance with ATS and ERS guidelines [16], and before starting the real study, we conducted a pilot study on 15 university students.

The required maneuver was demonstrated by the investigators, and subjects were encouraged and supervised throughout test performance. The test required the participant to assume a correct sitting upright posture; attach nose clip; place mouthpiece in the mouth and close lips around the mouthpiece; inspire completely and rapidly with a pause ≤ two seconds at TLC; and expire with a maximal effort until no more air can be expelled while maintaining an upright posture and for at least for 6 seconds; multiple trials were taken to ensure at least three acceptable and repeatable maneuvers, but not more than eight trials. The test was implemented at the university clinics where participants were tested in a closed room, and their privacy was ensured. The following spirometry parameters were included: FEV_1_, FVC, FEV_1_/FVC, PEF, and FEF_25–75_%.

A weighing scale and a meter for measuring weight and height were used. At last, an interviewer-administered questionnaire in Arabic language, pretested on a group of students, was administered to each participant for collecting the following variables: age, sex, duration of WPS in years, rate of WPS, and physical activity (150 minutes of the moderate-intensity aerobic physical activity or at least 75 minutes of the vigorous-intensity aerobic physical activity throughout the week or an equivalent combination of the moderate- and vigorous-intensity activity) [17].

### 2.3. Analysis Plan

The Statistical Package for Social Sciences (SPSS) software was used for data entry and analysis. Categorical characteristics were described using frequency tables. The average mean and standard deviation of spirometric values were computed, and the independent *t*-test and chi-squared test were used to assess the difference between the WP smoker and the nonsmoker groups. The multiple linear regression model was used to assess the relation between WPS and spirometry parameters while controlling for physical activity. The significance level was set at 0.05.

## 3. Results

The sample included 300 participants: 150 WP smokers and 150 nonsmokers. Age spanned from 18 to 26 with a mean age of 20.6 ± 1.7. The sex distribution in each group was 100 males and 50 females. Waterpipe smokers have smoked for an average of 3.8 (±1.6) years and with a frequency of 9.6 waterpipe heads per week. No significant difference was reported between the two groups (*P* value >0.05), except for the physical activity, which was significantly lower in the WP smoker group in general and among males (*P* value <0.001) (Table 1).

**Figure 1:**
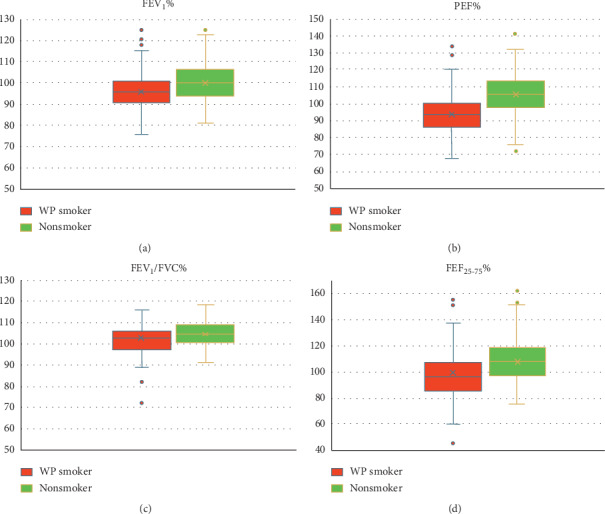
Comparison of spirometric parameters between waterpipe smokers and nonsmoker students. Values were presented as mean ± SD of FEV_1_%, PEF%, FEV_1_/FVC%, and FEF _25–75_% predicted.

The multiple linear regression model was used to assess the difference between the WP smoker and the nonsmoker groups' results taking into consideration the physical activity level among the participants. Significant differences regarding spirometry variables were observed between both groups (*P* < 0.05), except for FVC, with nonsmokers obtaining better results in FEV_1_, PEF, FEV_1_/FVC, and FEF _25–75_ (Table 2 and Figure 1). On the other hand, no significant correlation was observed between spirometric values and the amount of smoking (WPS heads per week and duration).

Table 3 compares the spirometric parameters between smokers and nonsmokers with male and female results presented separately. Considering the female students, the background variables were distributed equally between the WP smoker and nonsmoker groups. A significant decline in all spirometry parameters was reported in the WP smokers compared to the nonsmokers (*P* ≤ 0.001), except for FVC and FEV_1_. For male students, the WPS group was found to be significantly less physically active and reported significant decline in all spirometry parameters (*P* ≤ 0.001), except for FVC, compared to the nonsmoker group.

## 4. Discussion

Tobacco smoking is known to cause a myriad of respiratory diseases, both obstructive diseases (such as bronchitis, bronchiolitis, and chronic obstructive pulmonary disease) and restrictive diseases (smoking-related interstitial lung diseases), in addition to lung cancer. Waterpipe tobacco smoking is as addictive as regular tobacco use and is increasing in the Middle East and worldwide, especially among the youth. Many of them perceive it as a safer method of smoking. Because young WPS users are usually asymptomatic and do not seek medical care, few studies have addressed WPS effects on respiratory health in this population [8, 10, 11].

Our study demonstrates the effect of WPS on spirometry in a group of young asymptomatic individuals, with a relatively short period of WPS use. The results showed most spirometric parameters (FEV_1_, PEF, FEV_1_/FVC, and FEF_25–75_) to be lower in the smoking group. Mean parameter differences between the smoking and nonsmoking groups were all statistically significant (<0.001) except for FEV_1_ in females and FVC in both genders.

The most striking finding was a larger reduction in the midflow rate (FEF_25–75_%). Although this parameter is nonspecific and highly variable with less clinical relevance compared to other parameters (except PEF), midflows are effort independent and very sensitive for early airway and lung parenchymal disease. In other studies, the midexpiratory flow rate was found to drop significantly shortly after smoking WP [10]. This could possibly be an early predictor of small airway obstruction, and the implication of serial spirometry testing could then predict progression to full COPD [9].

Although reduced spirometric values of smokers were still in the normal range and no specific pattern of lung function impairment could be deduced from the mean values, this contrasting difference with the nonsmoking population inclines a possible start of the lung function deterioration.

Our results are comparable to other studies of young smokers from Jordan and Saudi Arabia, which showed reduced spirometric values among WPS compared to matched control groups [10, 11]. Additionally, Hawari et al. examined the effects of WPS on cardiopulmonary exercise (CPET) in a group of young healthy males. WP users had shorter exercise times, lower spirometric values, lower maximum oxygen consumption (VO_2_ ml/kg), lower HR and higher HR reserves, lower degree of change in end-expiratory lung volume (EELV), and higher PetCO_2_ and VE/VCO_2_ [10].

Studies by Boskabady et al., Ben Saad et al., and Kiter et al., which investigated older smoker age groups, demonstrated a consistent trend toward reduced spirometric values [12, 18, 19]. This is in line with our prediction of future decline in pulmonary function in the now-young smoking population.

Boksabady et al. (mean age 40.9) showed a significant negative correlation between the amount smoked and the reduction in PFTs, which was not statistically evident in our study [12]. This is perhaps due to the younger age of our participants and their supposedly more compliant lungs.

Female WP smoking students showed significant decrease in most spirometric parameters (FEV_1_/FVC%, PEF%, and FEF_25–75_%) compared to the nonsmokers. These findings are very important to consider, as it is the first study to include females in this age group, given the increasing rate of female WP smokers and the wrong perception of WP smoking to be less harmful in Palestine [4, 14]. The insignificant reduction of FEV1 in female smokers compared to males could be explained by the fact that females in our sample smoked less than males (8 vs 10.5 WP heads per week; *P* value = 0.001) and for a shorter duration (2.9 vs 4.2 years; *P* value <0.001).

In our study, factors such as age, BMI, and race/ethnicity were similar in both groups and were considered to have no or minor effects on results. Physical activity was lower in the WP smoker group (Table 1), which could be attributed to the characteristic WPS session that involves sitting for a considerable amount of time with access to food throughout the process. This surely facilitates a sedentary lifestyle [20].

### 4.1. Strengths and Limitations

To the best of our knowledge, this is the first study to assess spirometry parameters of young WP smokers, including both males and females, and with a relatively large sample size (300 university students). However, we merely tested WPS effects on lung function in terms of spirometry parameter and did not put into consideration clinical symptoms such as cough and exertional dyspnea that this young smoking population may complain of. Additionally, a nonprobability sampling technique was used, given the difficulty of accessing subjects fitting our inclusion criteria (regular waterpipe use for at least once a week for a one year).

## 5. Conclusion

Waterpipe smoking is associated with reduced spirometric values in apparently healthy young smokers with relatively limited smoking years. Incorporating serial spirometry into clinical practice could predict progression to COPD among young WP smokers. Emphasis on counseling young WP smokers for smoking cessation is important.

## Figures and Tables

**Table 1:** Baseline characteristics of sample of waterpipe smokers and nonsmokers (*n* = 300).

Characteristics	Total	WP smoker (*n* = 150)	Nonsmoker (*n* = 150)	*P* value^*∗*^
Age in years (mean ± SD)	20.6 ± 1.7	20.7 ± 1.7	20.5 ± 1.8	0.325

Weight in kg (mean ± SD)	72.4 (±14.6)	71.8 ± 14.4	73.0 ± 14.8	0.925

Height in cm (mean ± SD)	171.9 (±8.7	171.4 ± 8.7	172.5 ± 8.7	0.992

BMI	24.37 ± 3.9	24.35 ± 4.07	24.38 ± 3.73	0.963

Sex				
Male	200 (66.7%)	200 (66.7%)	100 (66.7%)	1.0
Female	100 (33.4%)	100 (33.4%)	50 (33.4%)	

Physical activity				
None	107 (35.7%)	70 (46.7%)	37 (24.7%)	
Sometimes	147 (49.0%)	66 (44.0%0	81 (54.0%)	<0.001
Regular	46 (15.3%)	14 (9.3%)	32 (21.3%)	

WPS heads per week (mean ± SD (range))	—	9.6 ± 6.6 (1–35)	—	—

Duration WPS in years (mean ± SD (range))	—	3.8 ± 1.6 (1-8)	—	—

^*∗*^Independent *t*-test and chi-squared test.

**Table 2:** Spirometry results comparing waterpipe smokers and nonsmokers

Parameters^¥^	WP smoker (*n* = 150)	Nonsmokers *(n* = 150)	*P* value^*∗*^
FEV_1_	3.96 (±0.7)	4.19 (±0.8)	<0.001
FEV_1_%	95.9 (±8.5)	100.5 (±9.3)	0.002
FVC	4.6 (±0.9)	4.7 (±0.9)	0.935
FVC%	95.0 (±8.8)	96.5 (±10.1)	0.194
PEF	8.4 (±1.8)	9.2 (±1.8)	0.002
PEF%	94.4 (±11.6)	104.4 (±12.0)	<0.001
FEV_1_/FVC	85.9 (±5.7)	88.7 (±4.8)	<0.001
FEV_1_/FVC%	101.5 (±6.8)	104.7 (±4.5)	0.002
FEF_25–75_	4.3 (±1.0)	4.9 (±1.0)	<0.001
FEF_25–75_%	96.2 (±18.7)	109.7 (±18.4)	<0.001

^¥^Values are presented in mean ± SD. ^*∗*^Adjusted *P* values were obtained using linear regression.

**Table 3:** Spirometry results comparing female and male waterpipe smokers and nonsmokers.

Parameters^¥^	Female			Male		
	WP smoker (*n* = 50)	Nonsmoker (*n* = 50)	*P* value^*∗*^	WP smoker (*n* = 100)	Nonsmokers (*n* = 100)	*P* value^*∗∗*^
FEV_1_	3.2 (±0.4)	3.3 (±0.5)	0.207	4.3 (±0.4)	4.6 (±0.5)	<0.001
FEV_1_%	96.2 (±8.8)	98.5 (±10.1)	0.216	95.7 (±8.4)	101.5 (±8.8)	<0.001
FVC	3.7 (±0.5)	3.6 (±0.5)	0.552	5.1 (±0.6)	5.3 (±0.6)	0.085
FVC%	96.4 (±8.6)	94.5 (±10.5)	0.319	94.2 (±8.9)	97.5 (±9.8)	0.047
PEF	6.4 (±1.0)	7.3 (±1.0)	<0.001	9.3 (±1.2)	10.2 (±1.2)	<0.001
PEF%	93.4 (±13.4)	105.2 (±13.3)	<0.001	94.9 (±10.6)	104.0 (±11.4)	<0.001
FEV_1_/FVC	87.6 (±4.1)	91.3 (±3.8)	<0.001	85.2 (±6.2)	87.5 (±4.8)	0.004
FEV_1_/FVC%	101.4 (±4.8)	105.6 (±4.5)	<0.001	101.6 (±7.7)	104.3 (±5.9)	0.006
FEF_25–75_	3.6 (±0.7)	4.2 (±0.8)	0.001	4.6 (±0.9)	5.3 (±0.9)	0.001
FEF_25–75_%	97.1 (±16.7)	112.3 (±20.0)	<0.001	95.8 (±19.7)	108.4 (±17.5)	<0.001

^¥^Values are presented in mean ± SD. ^*∗*^Independent *t*-test. ^*∗∗*^Adjusted *P* values were obtained using linear regression.

## Data Availability

The data used to support the findings of this study are available from the corresponding author upon request.
